# Eicosapentaenoic acid exhibits antitumor effects and synergistically enhances carboplatin sensitivity in endometrial cancer

**DOI:** 10.3389/fphar.2026.1960634

**Published:** 2026-10-21

**Authors:** Haomeng Zhang, Weimin Kong, Shuning Chen, Xiaochang Shen, Alexandra Diggs, Miller Singleton, Boer Deng, Chelsey Vranes, Glenn Boyles, Chunxiao Zhou, Victoria Bae-Jump

**Affiliations:** 1 Department of Gynecology, Beijing Obstetrics and Gynecology Hospital, Capital Medical University. Beijing Maternal and Child Health care Hospital, Beijing, China; 2 Division of Gynecologic Oncology, University of North Carolina at Chapel Hill, Chapel Hill, NC, United States; 3 Lineberger Comprehensive Cancer Center, University of North Carolina at Chapel Hill, Chapel Hill, NC, United States

**Keywords:** apoptosis, eicosapentaenoic acid (EPA), endometrial cancer, invasion, synergy

## Abstract

**Background:**

Obesity-related metabolic dysfunction and chronic inflammation are well-established drivers of the carcinogenesis and progression of endometrial cancer (EC). Eicosapentaenoic acid (EPA) is a long-chain omega-3 polyunsaturated fatty acid that has previously demonstrated broad anti-inflammatory and anti-tumor properties in various cancer models. In this study, we evaluated the anti-tumorigenic effects of EPA in EC cell lines and in a genetically engineered mouse model of endometrioid EC.

**Methods:**

Human EC cell lines, KLE and HEC-1B, were used to evaluate the effects of EPA alone or in combination with carboplatin on cell proliferation, invasion, cell cycle profile, stress response, and apoptosis. An *LKB1* ^*fl/fl*^ *p53* ^*fl/fl*^ genetically engineered mouse model of endometrioid EC was used to examine the effects of EPA on endometrial tumor growth.

**Results:**

EPA treatment markedly suppressed cell proliferation and colony formation in a dose-dependent manner. Mechanistically, EPA induced G1 cell cycle arrest, promoted cellular stress, and activated cleaved caspases, while inhibiting cell adhesion and migration. These phenotypes were accompanied by robust inhibition of the AKT/mTOR signaling axis and activation of MAPK-mediated stress pathways. Inflammatory signaling was similarly attenuated, as evidenced by reduced TNF-α–induced COX-2 expression and decreased phosphorylation of NF-κB. Importantly, EPA synergized with carboplatin to enhance anti-tumor efficacy. Combination treatment produced stronger suppression of cell proliferation and invasion, increased apoptotic activity, and downregulation of key survival and oncogenic regulators, including phosphorylated AKT, Bcl-2, β-catenin, and CDK4. Four weeks of EPA administration significantly reduced endometrial tumor burden in both obese and lean mice, accompanied by decreased expression of Ki-67, Bcl-xL, β-catenin, and COX-2 in endometrial tumor tissues.

**Conclusion:**

EPA exerts potent anti-tumor activity in EC through coordinated modulation of oncogenic signaling, inflammatory pathways, cellular stress responses, and apoptosis. Moreover, its ability to enhance carboplatin sensitivity highlights the potential of EPA as an adjuvant therapeutic candidate for EC and warrants further investigation in preclinical models of EC.

## 1 Introduction

Endometrial cancer (EC) is the most common gynecological malignancy in developed countries, and its global incidence continues to rise. In the United States alone, projections for 2026 estimate approximately 68,270 new cases of uterine corpus cancer and 14,450 related deaths (Siegel et al., 2026). Although well-established risk factors, such as obesity, diabetes, chronic estrogen exposure, nulliparity, and persistent inflammation, contribute substantially to disease development, emerging evidence highlights metabolic reprogramming as a central driver of EC pathogenesis. In particular, dysregulated lipid metabolism has been strongly implicated in promoting tumor progression and fostering more aggressive biological behavior (Wang et al., 2024; Hooks et al., 2025). While early-stage EC generally carries a favorable prognosis, advanced and recurrent disease remains a major clinical challenge (Hooks et al., 2025; Buza et al., 2026). These high-risk tumors frequently acquire resistance to conventional hormonal therapies, cytotoxic chemotherapy, and targeted agents, contributing significantly to rising mortality rates (Buza et al., 2026). Consequently, there is an urgent need to elucidate the molecular mechanisms underlying these metabolic alterations to identify novel therapeutic vulnerabilities and develop more effective treatment strategies (Ronsini et al., 2024; Pados et al., 2023).

Long-chain omega-3 (n-3) polyunsaturated fatty acids (PUFAs), including eicosapentaenoic acid (EPA) and docosahexaenoic acid (DHA), are essential bioactive lipids required for maintaining physiological homeostasis. Because endogenous conversion from α-linolenic acid (ALA) is limited, these fatty acids are obtained primarily from marine sources (Calder, 2017; Burdge and Calder, 2005). Extensive evidence demonstrates that EPA and DHA exert diverse biological functions, ranging from anti-inflammatory activity and cardiovascular protection to regulation of neural development, metabolic homeostasis, immune responses, reproductive health, and anti-cancer effects, largely through their roles in membrane remodeling, cell-signaling modulation, and the generation of specialized lipid mediators (Calder, 2017; Mozaffarian and Rimm, 2006; King et al., 2025; Molfino et al., 2026). Epidemiological studies consistently report that higher dietary intake of EPA and DHA is associated with reduced risk of several malignancies, including breast, colorectal, and prostate cancers (Caini et al., 2022; Zheng et al., 2013). Supporting these observations, animal studies show that diets enriched with EPA and DHA decrease tumor incidence and tumor burden in chemically induced models of colon, breast, and liver cancer, as well as in xenograft mouse models (Caini et al., 2022; Ljungblad et al., 2022; Rahman et al., 2013). Notably, EPA appears to exert a more pronounced influence than DHA on inflammatory signaling and lipid-mediator biosynthesis, making it particularly relevant in diseases driven by chronic inflammation, such as cancer and metabolic disorders (Calder, 2017; Calder, 2018). Although clinical evidence remains limited regarding whether EPA reduces EC risk or improves prognosis, emerging preclinical data are promising. A recent study demonstrated that EPA ameliorates estradiol valerate-induced endometrial hyperplasia at both biochemical and histopathological levels, likely through modulation of the NF-κB/HIF-1α/VEGF signaling pathway in rats (Abdelzaher et al., 2023; Brasky et al., 2023; Arem et al., 2013). These findings suggest that EPA may possess meaningful anti-tumor activity in the prevention and potential treatment of endometrial cancer.

EPA is a pleiotropic bioactive lipid that has been approved by the FDA for the treatment of severe hypertriglyceridemia and for reducing cardiovascular risk in high-risk patients (Boden et al., 2021). Beyond its established clinical applications, extensive preclinical evidence demonstrates that EPA exerts potent anti-inflammatory and anti-cancer activities across multiple tumor models. Given the central roles of chronic inflammation and metabolic dysregulation in the carcinogenesis and progression of EC (Tan et al., 2025; Irún et al., 2019), we investigated the potential anti-tumor effects of EPA in EC cell lines and in an *LKB1* ^*fl/fl*^ *p53* ^*fl/fl*^ genetically engineered mouse model of endometrioid EC. Our findings show that EPA treatment attenuated the inflammatory response, suppressed cell proliferation, induced apoptosis, and inhibited tumor growth. Moreover, EPA enhanced the therapeutic efficacy of carboplatin, exhibiting synergistic anti-tumor activity in EC cell lines. Together, these results highlight EPA as a promising dietary candidate for further exploration as an adjuvant therapeutic strategy in EC.

## 2 Materials and methods

### 2.1 Cell culture and reagents

Human endometrial cancer cell lines RL95-2, AN3CA, KLE, and HEC-1B were obtained from the American Type Culture Collection, and the EC-023 cell line was generously provided by Dr. Gertz (University of Utah School of Medicine). All cell lines were routinely authenticated by short tandem repeat (STR) profiling (LabCorp, Burlington, NC) during the course of the study. KLE and RL95-2 cells were cultured in DMEM/F12 supplemented with 10% fetal bovine serum (FBS; Phoenix Scientific, St. Joseph, MO). HEC-1B cells were maintained in McCoy’s 5A medium with 10% FBS, Ishikawa cells in MEM containing 5% FBS, EC-023 cells in RPMI-1640 with 10% FBS, and AN3CA cells in DMEM supplemented with 10% FBS. All media were supplemented with 100 U/mL penicillin and 100 μg/mL streptomycin, and cells were maintained at 37 °C in a humidified incubator with 5% CO_2_. Analytical-grade EPA (No. 90110, purity ≥99%) was purchased from Cayman Chemical (Ann Arbor, MI). 3-(4,5-Dimethylthiazol-2-yl)-2,5-diphenyltetrazolium bromide (MTT, Sigma-Aldrich, M5655), dimethyl sulfoxide (DMSO, Fisher Scientific, BP231-1), and additional chemical reagents were obtained from Thermo Fisher Scientific (Waltham, MA) and Sigma-Aldrich (St. Louis, MO). All antibodies used in Western blotting and immunohistochemistry were purchased from Cell Signaling Technology (Danvers, MA, USA), including) AKT (Cat. No. 9272), phospho-AKT^S473^ (Cat. No. 4060), phospho-S6 (Cat. No. 4858), S6 (Cat. No. 2217), phospho-ERK1/2(Cat. No.9101), ERK1/2(Cat. No.4695), PERK (Cat. No.5683), PDI (Cat. No.3501), Calnexin(Cat. No.2679), CDK2(Cat. No.18048), CDK4(Cat. No.12790), Cyclin D1(Cat. No.55506), Bcl-xL(Cat. No.2764), Bcl-2(Cat. No.3498), Mcl-1(Cat. No.94296), Bax(Cat. No.5023), β-catenin(Cat. No.8480), Snail (Cat. No.3879), Slug (Cat. No.9585), Cox-2 (Cat. No.12282), phospho-NF-kappaB p65(Cat. No.3033), NF-kappaB p65(Cat. No.8242) and α-Tubulin(Cat. No. 2144) were used in this study. Flurogenic ELISA substrates Ac-DEVD AMC (Catalog No.: 13402), Ac-IETD-AFC (Catalog No.: 13410), and Ac-LEHD-AMC (Cat. No. 13426) were purchased from AAT Bioquest (Pleasanton, CA).

### 2.2 Cell proliferation assay

Cells were enzymatically dissociated into single-cell suspensions, and 4,000-6,000 cells per well were reseeded into 96-well plates. After a 24-h attachment period, cells were treated with EPA across a concentration range of 0.1–500 µM. Following an additional 72-h incubation, cell viability was assessed using the MTT assay. Briefly, 5 µL of 5 mg/mL MTT solution was added to each well and incubated for 1 h at 37 °C. The supernatant was then aspirated, and 100 µL DMSO was added to solubilize the formazan crystals. Absorbance was measured at 562 nm using a Thermo microplate reader (Cleveland, OH). All measurements were performed in triplicate. Viability values were normalized to untreated controls, and dose-response curves were generated to calculate IC50 values (AAT Bioquest. Pleasanton, CA). The Bliss independence model was used to evaluate drug interactions, with CI < 1 indicating synergy, CI = 1 indicating an additive effect, and CI > 1 indicating antagonism.

### 2.3 Colony assay

For the colony formation assay, 400-600 cells per well were plated in six-well plates. After an attachment period, the cells were treated with EPA at concentrations of 10, 50, and 100 μM for 36 h. The cells were allowed to colonize for 12–14 days. To visualize the colonies, the culture medium was removed, and cells were washed twice with PBS. The plates were then stained with 0.3% crystal violet staining solution for 30 min and rinsed with tap water for 30 min. The plates were scanned, and masses exceeding 50 cells were regarded as colonies.

### 2.4 Cell cycle assay

Image flow cytometry was used to investigate the impact of EPA treatment on cell cycle progression. KLE and HEC-1B cells were seeded into 6-well plates at a density of 4–5 × 10^5^ cells per well and cultured overnight. Cells were treated with EPA at concentrations of 10, 50, and 100 μM for 24 h. After treatment, the cells were washed, detached and fixed with methanol on ice for 30 min. The fixed cells were then suspended in 500 µL of PBS containing 5 µL of RNase A and PI and incubated for 30 min at 37 °C. Cellometer (Nexcelom, Lawrence, MA) was used to identify the cell cycle profile, and FCS7 Express (Molecular Devices, Sunnyvale, CA) software was used to analyze the data.

### 2.5 Reactive oxygen species (ROS) assay

Cellular ROS level was measured using 2ʹ,7ʹ-dichlorofluorescin diacetate (DCFDA) assay. KLE and HEC-1B cells were seeded into 6-well cell culture plates and incubated for 24 h. Subsequently, 10, 50, and 100 μM of EPA were added, and cells were treated for 3 h. Following indicated treatments, cells were stained in the culture medium with 10 µM DCFDA at 37 °C for 30 min. After washing twice with PBS, the fluorescence intensity was detected by a Thermo plate reader at 485 nm excitation (Ex) and 525 nm emission (Em) wavelengths.

### 2.6 Mitochondrial membrane potential assay

Changes in mitochondrial membrane potential were assessed using a JC-1-based ELISA assay. Cells were plated in 96-well plates at a density of 30,000 cells/well overnight in complete medium. Then cells were treated with 10, 50, and 100 μM EPA for 3 h. Cells were stained with 2 μM JC-1 solution for 30 min at 37 °C in the dark. Cells were measured and analyzed at wavelengths Ex/Em 485/535 nm by a Thermo microplate reader.

### 2.7 Cleaved caspase 3, 8, and 9 ELISA assays

After 3 h of exposure to 10, 50, and 100 μM EPA, KLE and HEC-1B cells were scraped and collected using cell lysis buffer. The cell lysates were mixed with the appropriate cleaved caspase substrates and DTT in reaction buffer for 30 min in the dark. The mixtures were transferred to a black, clear-bottom 96-well plate, and fluorescence was measured using a Thermo microplate reader. Cleaved caspase-3 and caspase-9 were detected at Ex/Em 341/441 nm, and cleaved caspase-8 at Ex/Em 376/482 nm.

### 2.8 Western blotting assay

Cells were lysed in RIPA Buffer, and protein concentrations of the cell lysates were determined with BCA assay (Thermo Fisher Scientific). Protein lysates were then subjected to 10%–12% Tris-Glycine SDS PAGE and then transferred onto PVDF membranes. The membranes were blocked in 5% milk-Tris-buffered saline with 0.1% Tween (TBST) at 25 °C for 1 h and incubated with primary antibodies at 4 °C overnight. After washing three times with TBST, the membrane was incubated with a goat anti-rabbit or goat anti-mouse secondary antibody for 1 h. Finally, the proteins were detected by Western Lightning Plus-ECL (PerkinElmer) on the ChemiDoc Image System with Image Lab software (version 6.1, Bio-Rad). ImageJ was used to analyze the integrated density values.

### 2.9 Adhesion assay

The 96-well plates were coated with PBS (as a negative control) and 10 μg/mL laminin and incubated overnight at 4 °C. To block any remaining protein binding sites on the plate, coating solutions were removed, and 5% bovine serum albumin (BSA) in PBS was added for another 30 min. KLE and HEC-1B cell suspensions at a density of 2 × 10^5^ cells/mL were co-inoculated with varying doses of EPA, followed by continued incubation for 1.5 h. Non-adherent cells were removed by careful washing with PBS once, and 100 µL 5% glutaraldehyde per well was added into the plates for 30 min followed by 0.1% crystal violet staining for another 15 min. After excess stain was removed, the plates were solubilized with 100 µL acetic acid. The absorbance was measured at 570 nm using a Thermo microplate reader. Experiments were repeated three times with four replications per experiment.

### 2.10 Wound healing assay

KLE and HEC-1B cells (5-6 x 10^5^ cells per well) were seeded on 6-well plates and cultured to near 80%–90% confluence. The monolayers were scratched with a sterile pipette tip, and the floating cells were removed with PBS. The cells were then treated with EPA for another 24 or 48 h, and the images of the wound gap were recorded under an inverted microscope at ×40 magnification. The migration ability was quantified by calculating the scratch-healing rate.

### 2.11 *Lkb1* ^*fl/fl*^ *p53* ^*fl/fl*^ transgenic mouse model of EC

The *LKB1* ^*fl/fl*^ *p53* ^*fl/fl*^-genetically engineered mouse model of endometrioid EC was generated in our laboratory (Guo et al., 2019). Because EC is a highly obesity-driven cancer, we evaluated the effects of EPA on tumor growth in both obese and lean mice. Four-week-old mice were randomly assigned to two dietary groups: a high-fat diet (HFD, obese group; 60% of calories from fat; Research Diets, New Brunswick, NJ) to induce obesity, or a low-fat diet (LFD, lean group; 10% kcal from fat; Research Diets) to maintain lean body weight. Recombinant adenovirus Ad5-CMV-Cre (Ad-Cre, Transfer Vector Core, University of Iowa), at a titer of 10^11^–10^12^ infectious particles/mL, was used for intrauterine delivery to induce tumor formation. At 8 weeks of age, Ad-Cre was injected into the left uterine horn of *LKB1* ^*fl/fl*^ *p53* ^*fl/fl*^ mice. Following tumor induction, mice were randomly divided into four subgroups: HFD-control, HFD-EPA, LFD-control, and LFD-EPA, with at least 15 mice per subgroup. Mice in the obese group had a significantly higher average body weight than those in the lean group (44.29 g vs. 26.46 g), EPA treatment (50 mg/kg, by gavage) was administered every other day for 4 weeks, beginning 8 weeks after Ad-Cre injection. Body weight was recorded weekly. Throughout the treatment period, the mice exhibited normal behavior and physical activity, with no obvious signs of toxicity or adverse effects. Endometrial tumors were collected and weighed; half of each tumor was fixed in 10% neutral-buffered formalin and paraffin-embedded, while the remaining half was snap-frozen and stored at −80 °C. All animal procedures were approved by the Institutional Animal Care and Use Committee (IACUC, 24-161) at the University of North Carolina at Chapel Hill.

### 2.12 Immunohistochemistry (IHC) of endometrial tumors

After overnight fixation in 10% formalin, paraffin-embedded tissue blocks were sectioned at 5 μm using a rotary microtome. Slides were deparaffinized in xylene and rehydrated through a graded ethanol series. Antigen retrieval was performed by boiling the slides in fresh retrieval buffer for 3 min in a pressure cooker, followed by cooling in cold water for 10 min. To block nonspecific binding, sections were incubated with a protein block solution (Dako, Agilent Technologies, Santa Clara, CA) for 1 h at room temperature and then incubated overnight at 4 °C with primary antibodies purchased from Cell Signaling Technology against Ki-67(Cat.No. 9027, 1:800), Bcl-xL(Cat. No.2764, 1:600), p-S6 (Cat.No.4858, 1:1200), and Cox-2 (Cat.No.12282, 1:300). After PBS washes, slides were incubated with a horseradish peroxidase-conjugated secondary antibody at 37 °C for 30 min. Signal detection was performed using 3,3′-diaminobenzidine, followed by hematoxylin counterstaining. All IHC slides were scanned using a Motic imaging system (Houston, TX) and quantified using ImagePro 10 software (Vista, CA). Protein expression levels were categorized accordingly, and H-scores were calculated using the formula: H-score = (percentage of strong positive cells × 3) + (percentage of positive cells × 2) + (percentage of weak positive cells × 1).

### 2.13 Statistical analysis

All statistical analyses were performed using GraphPad Prism 10.0 (GraphPad Software, San Diego, CA). All *in vitro* experiments were independently repeated three times, and the results from the three independent experiments were used for statistical analysis (n = 3). Technical replicates within each independent experiment were averaged and were not treated as independent biological replicates. Data are presented as mean ± standard deviation (SD). For comparisons between two groups, an unpaired two-tailed Student’s t-test was used. For comparisons among multiple groups, one-way or two-way analysis of variance (ANOVA) was performed as appropriate, followed by the corresponding multiple-comparison test. A P value <0.05 was considered statistically significant. Statistical significance was defined as *p < 0.05 and **p < 0.01. #p < 0.05, ##p < 0.01, between groups.

## 3 Results

### 3.1 EPA inhibits cell proliferation and tumor growth in EC

To evaluate the effects of EPA on cell growth, five EC cell lines, including AN3CA, EC023, HEC-1B, KLE, and RL95-2, were treated with increasing concentrations of EPA for 72 h. MTT assays demonstrated that EPA significantly inhibited cell proliferation in all 5 cell lines in a dose-dependent manner, with half-maximal inhibitory concentration (IC50) values of 57.71 μM for AN3CA, 49.49 μM for EC023, 43.58 μM for HEC-1B, 88.63 μM for KLE, and 104.91 μM for RL95-2 cells (Figure 1A). To assess longer-term effects on cell viability, HEC-1B and KLE cells were treated with EPA for 36 h and subsequently cultured for an additional 12–14 days to evaluate colony formation. EPA markedly reduced clonogenic survival in both cell lines. At 50 μM, EPA decreased colony formation by 40.48% in HEC-1B cells and 35.14% in KLE cells (Figure 1B). Given the central role of the PI3K/AKT/mTOR pathway in EC pathogenesis, we next examined the effects of EPA on the phosphorylation of AKT, S6, and ERK1/2. Following 24 h of EPA treatment, both HEC-1B and KLE cells exhibited reduced phosphorylation of AKT and S6. Unexpectedly, EPA increased ERK1/2 phosphorylation in both cell lines (Figure 1C), suggesting activation of a compensatory MAPK-mediated stress response.

**FIGURE 1:**
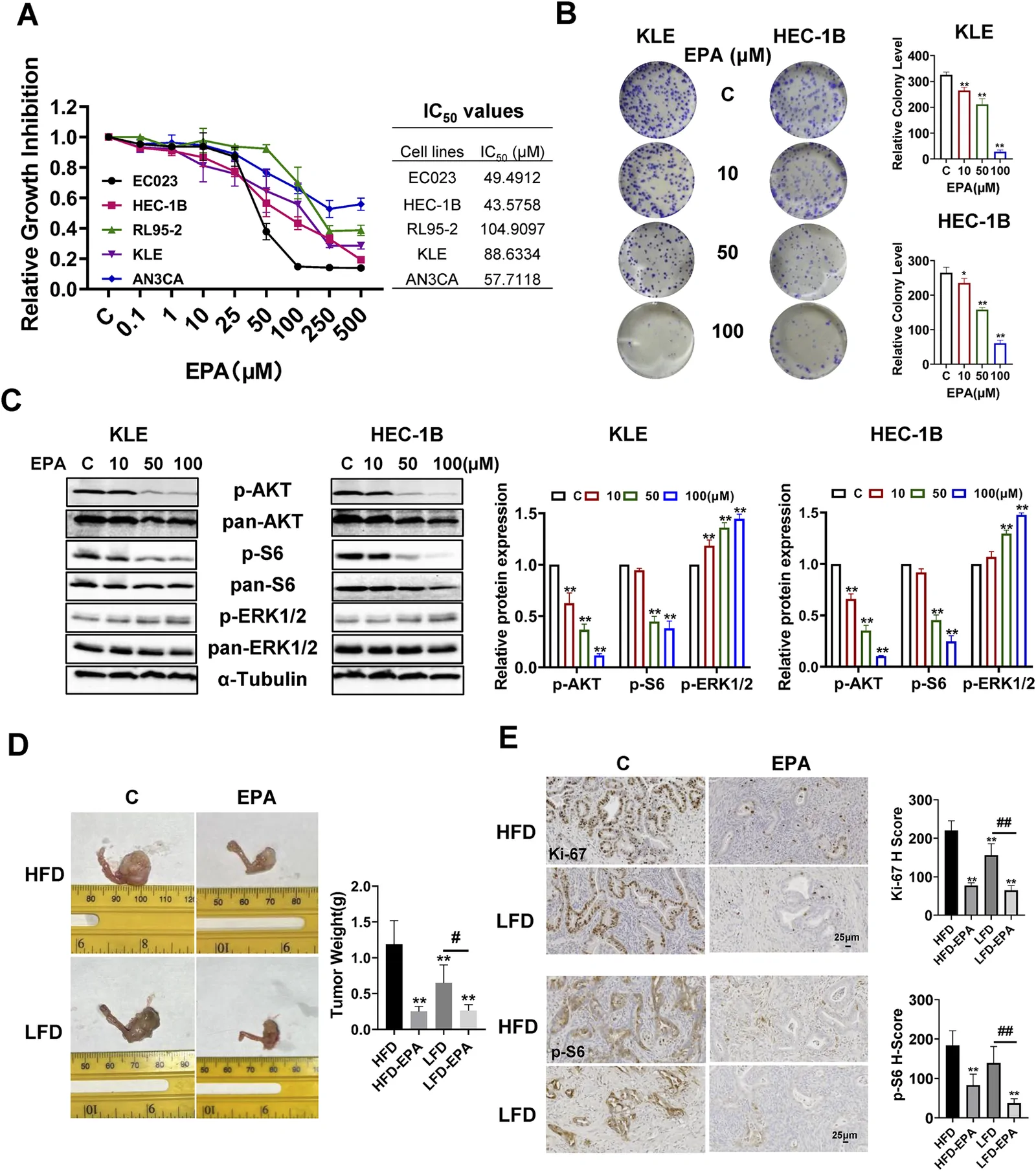
EPA inhibited cell proliferation and tumor growth. AN3CA, EC023, HEC-1B, KLE, and RL95-2 cells were treated with various concentrations of EPA for 72 h. MTT results demonstrated that EPA inhibited cell proliferation in all 5 cell lines. **(A)** KLE and HEC-1B cells were co-incubated with 10, 50, and 100 μM EPA for 36 h. Subsequently, cells were cultured for an additional 12–14 days. Colony formation assays revealed that EPA significantly inhibited colony formation in both KLE and HEC-1B cells in a dose-dependent manner. **(B)** Western blot analysis showed that following EPA treatment of KLE and HEC-1B cells for 24 h, EPA reduced expression of phosphorylated AKT and phosphorylated S6 and increased expression of phosphorylated ERK1/2. **(C)** In obese (HFD) and lean (LFD) *LKB1* ^*fl/fl*^ *p53* ^*fl/fl*^ mice, following 4 weeks of treatment, EPA effectively reduced the weight of endometrial tumors. **(D)** IHC results showed that EPA treatment significantly reduced the expression levels of Ki-67 and phosphorylated S6 in the endometrial tumor tissues of obese (HFD) and lean (LFD) mice. **(E)** *p < 0.05, **p < 0.01 compared with control. #p < 0.05, ##p < 0.01, between groups.

To determine whether these effects translated *in vivo*, EPA was administered continuously for 4 weeks to obese and lean *LKB1* ^*fl/fl*^ *p53* ^*fl/fl*^ mice. Compared with the control group, EPA treatment neither altered body weight nor reduced perimetrial fat weight in either obese or lean mice, nor did it cause apparent toxicity (Supplemental Figure). EPA significantly inhibited tumor growth under both metabolic conditions, with a more pronounced effect in obese mice, where tumor weight was reduced by 78.84%, compared with 57.70% reduction in lean mice (Figure 1D). IHC analysis further showed that EPA decreased Ki67 expression and S6 phosphorylation in endometrial tumor tissues from both groups (Figure 1E). Collectively, these findings indicate that EPA suppresses EC cell proliferation and tumor growth through modulation of the AKT/mTOR pathway and activation of MAPK-associated stress signaling, supporting its potential as a therapeutic agent in EC.

### 3.2 EPA induces cellular stress in EC

Given that EPA frequently exhibits a pro-oxidant effect in cancer cells (Fukui et al., 2013), we next examined its impact on ROS generation and mitochondria membrane potential in EC cells. Using the DCFDA assay, we quantified intracellular ROS levels following 3 h of EPA treatment across a range of concentrations in HEC-1B and KLE cells. EPA increased ROS production in a dose-dependent manner in both cell lines; specifically, 50 μM EPA elevated ROS levels by 10.55% in HEC-1B cells and by 12.46% in KLE cells (Figure 2A). Consistent with these findings, EPA significantly reduced mitochondrial membrane potential after 3 h of treatment. At 50 μM, EPA decreased mitochondrial membrane potential by 7.54% in HEC-1B and by 13.23% in KLE cells (Figure 2B). Western blot analysis further demonstrated that 2-4 h EPA treatment increased the expression of the endoplasmic reticulum (ER) stress markers PERK, PDI, and Calnexin in both cell lines (Figure 2C). Together, these results indicate that EPA exerts a pronounced pro-oxidant effect in EC cells, promoting ROS accumulation, mitochondrial depolarization, and activation of ER stress pathways.

**FIGURE 2:**
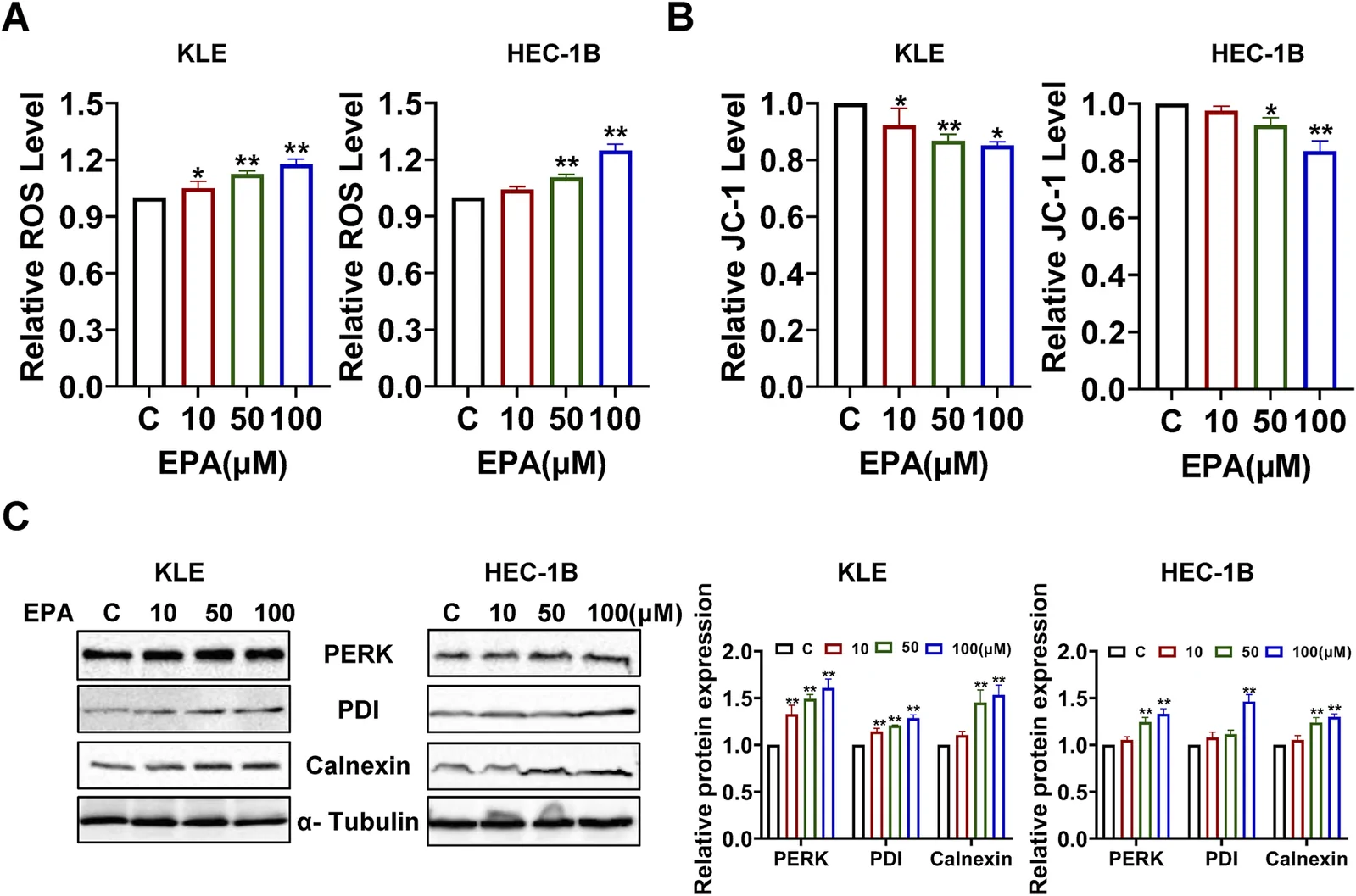
EPA induced cell stress in EC cells. KLE and HEC-1B cells were treated with increasing concentrations of EPA for 3 h, and DCFH-DA assays demonstrated that EPA induced a dose-dependent increase in intracellular ROS levels. **(A)** EPA reduced mitochondrial membrane potential following 3 h of treatment in KLE and HEC-1B cells. **(B)** Western blot analysis confirmed that EPA increased the expression of PERK, PDI, and Calnexin after 2-4 h of treatment in KLE and HEC-1B cells. **(C)** *p < 0.05, **p < 0.01 compared with control.

### 3.3 EPA causes cell cycle G1 arrest in EC

To determine whether EPA alters cell-cycle progression in EC cells, HEC-1B and KLE cells were treated with 10, 50, and 100 μM EPA for 24 h, followed by cell-cycle analysis using a Cellometer. EPA induced a dose-dependent G1-phase arrest in both cell lines. Treatment with 100 μM EPA increased the proportion of HEC-1B cells in G1 from 57.40% to 79.60% and increased the proportion of KLE cells in G1 from 54.43% to 74.28% (Figure 3A). Consistent with these findings, Western blot analysis revealed that EPA reduced the expression of key G1-regulatory proteins, including CDK2, CDK4, and Cyclin D1, after 24 h of treatment in both cell lines (Figure 3B). Collectively, these results demonstrate that EPA effectively induces G1-phase cell-cycle arrest in EC cells.

**FIGURE 3:**
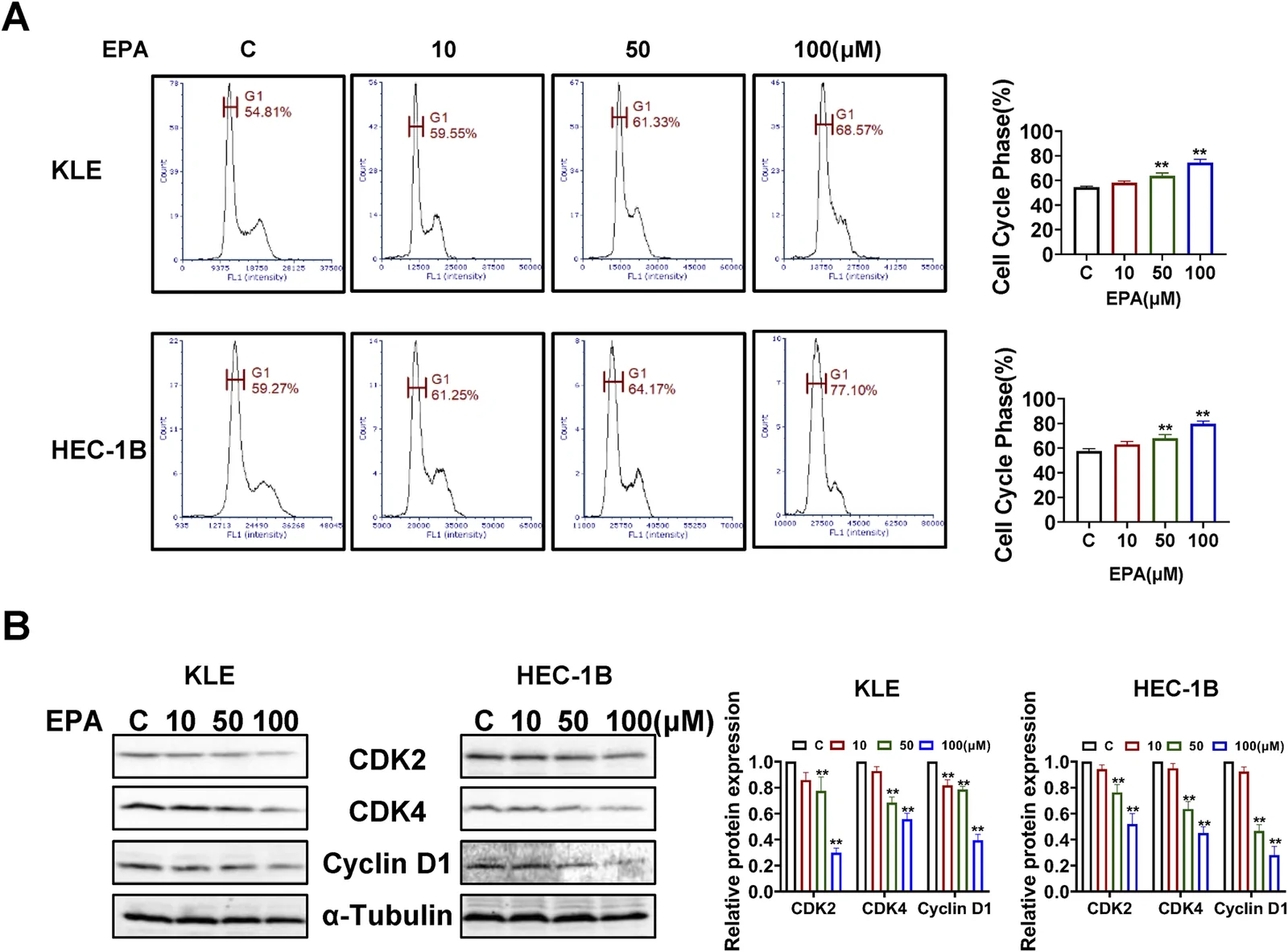
EPA caused G1 cell cycle arrest in EC cells. KLE and HEC-1B were treated with 10, 50, and 100 μM EPA for 24 h. Cell cycle profiles were analyzed using a Cellometer. The results demonstrated that EPA induced G1-phase cell-cycle arrest in a dose-dependent manner. **(A)** Western blot analysis exhibited that EPA inhibited the expression of CDK2, CDK4, and CyclinD1 in both cell lines after 24 h of treatment. **(B)** *p < 0.05, **p < 0.01 compared with control.

### 3.4 EPA induced apoptosis in EC

Because EPA induces G1-phase arrest and cellular stress, we next examined whether EPA suppresses EC cell proliferation by promoting apoptosis. HEC-1B and KLE cells were treated with 10, 50, and 100 uM EPA for 3 h, and caspase activity was quantified using ELISA-based assays. EPA significantly increased the activities of cleaved caspase-3, -8 and -9 in a dose-dependent manner in both cell lines. Specifically, 100 μM EPA elevated caspase-3, -8, and -9 activities by 37.70%, 23.42%, and 20.51%, respectively, in HEC-1B cells, and by 144.57%, 14.66%, and 74.50%, respectively, in KLE cells (Figures 4A–C). Consistent with these findings, Western blot analysis revealed that EPA treatment for 3–8 h decreased the expression of the anti-apoptotic proteins Bcl-2, Bcl-xL, and Mcl-1, while increasing the expression of the pro-apoptotic protein Bax in both cell lines (Figure 4D). Immunohistochemical (IHC) analysis further supported these *in vitro* observations: EPA treatment for 4 weeks reduced Bcl-xL expression in endometrial tumors from both obese and lean *LKB1* ^*fl/fl*^ *p53* ^*fl/fl*^ mice (Figure 4E). Together, these results demonstrate that EPA promotes apoptosis in EC cells through activation of both the intrinsic apoptotic pathway and the extrinsic apoptotic pathway, contributing to its overall anti-tumor activity.

**FIGURE 4:**
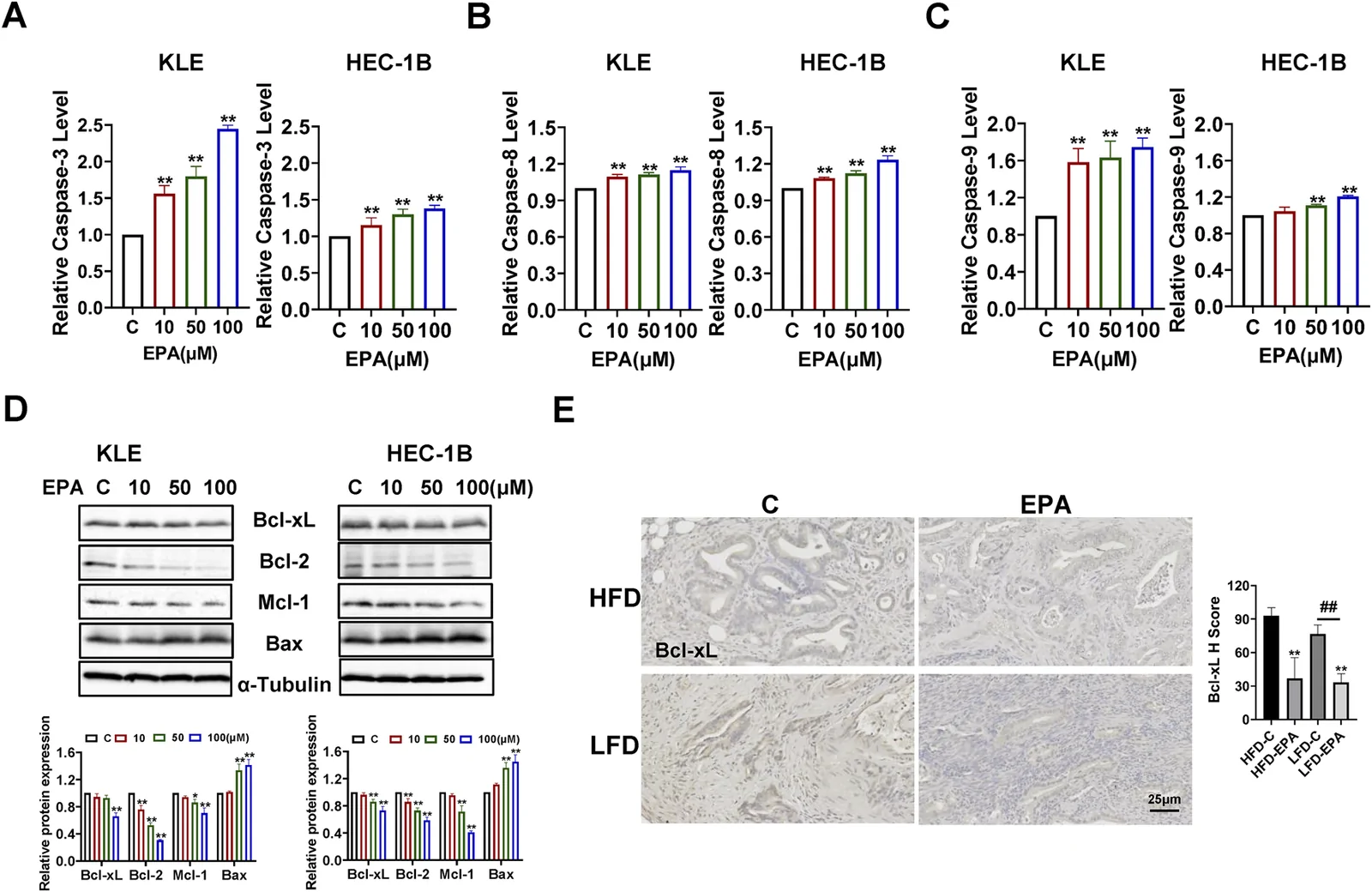
EPA induced apoptosis in EC cells. KLE and HEC-1B cell were treated with increasing concentrations of EPA for 3 h, and ELISA results demonstrated that EPA increased the activity of cleaved caspase-3, -8, and -9 in a dose-dependent manner. **(A–C)** Western blot analysis demonstrated that, following treatment of cells with increasing doses of EPA for 3–8 h, Bcl-xL, Bcl-2, and Mcl-1 expression levels were downregulated. **(D)** Following 4 weeks of EPA treatment, Bcl-xL expression was significantly decreased in the endometrial tumor tissues of both obese and lean *LKB1* ^*fl/fl*^ *p53* ^*fl/fl*^ mice. **(E)** *p < 0.05, **p < 0.01 compared with control. #p < 0.05, ##p < 0.01, between groups.

### 3.5 EPA reduced cell adhesion and migration in EC

Given that EPA has demonstrated anti-invasive activity in multiple cancer models, we next examined its effects on the invasive behavior of EC cells using adhesion and migration assays. Following treatment of HEC-1B and KLE cells with 10, 50, and 100 μM EPA for 90 min, adhesion assays showed that EPA reduced the adhesive capacity of both cell lines. Treatment with 50 μM EPA decreased adhesion by 10.10% in HEC-1B cells and by 11.51% in KLE cells (Figure 5A). Migration assays further demonstrated that EPA impaired cell motility in a dose-dependent manner; specifically, 50 µM EPA increased wound-healing width by 1.53-fold in KLE cells and 1.45-fold in HEC-1B cells, indicating reduced migratory potential (Figure 5B). To determine whether epithelial–mesenchymal transition (EMT) contributes to EPA-mediated inhibition of invasion, both cell lines were treated with 10, 50, and 100 μM EPA for 24 h. Western blot analysis revealed that EPA decreased the expression of EMT-associated proteins, including β-catenin, Slug, and Snail, in both cell lines (Figure 5C). Together, these findings indicate that EPA suppresses EC cell invasion, at least in part through inhibition of EMT-associated signaling pathways, thereby reducing cellular adhesion and migratory capacity.

**FIGURE 5:**
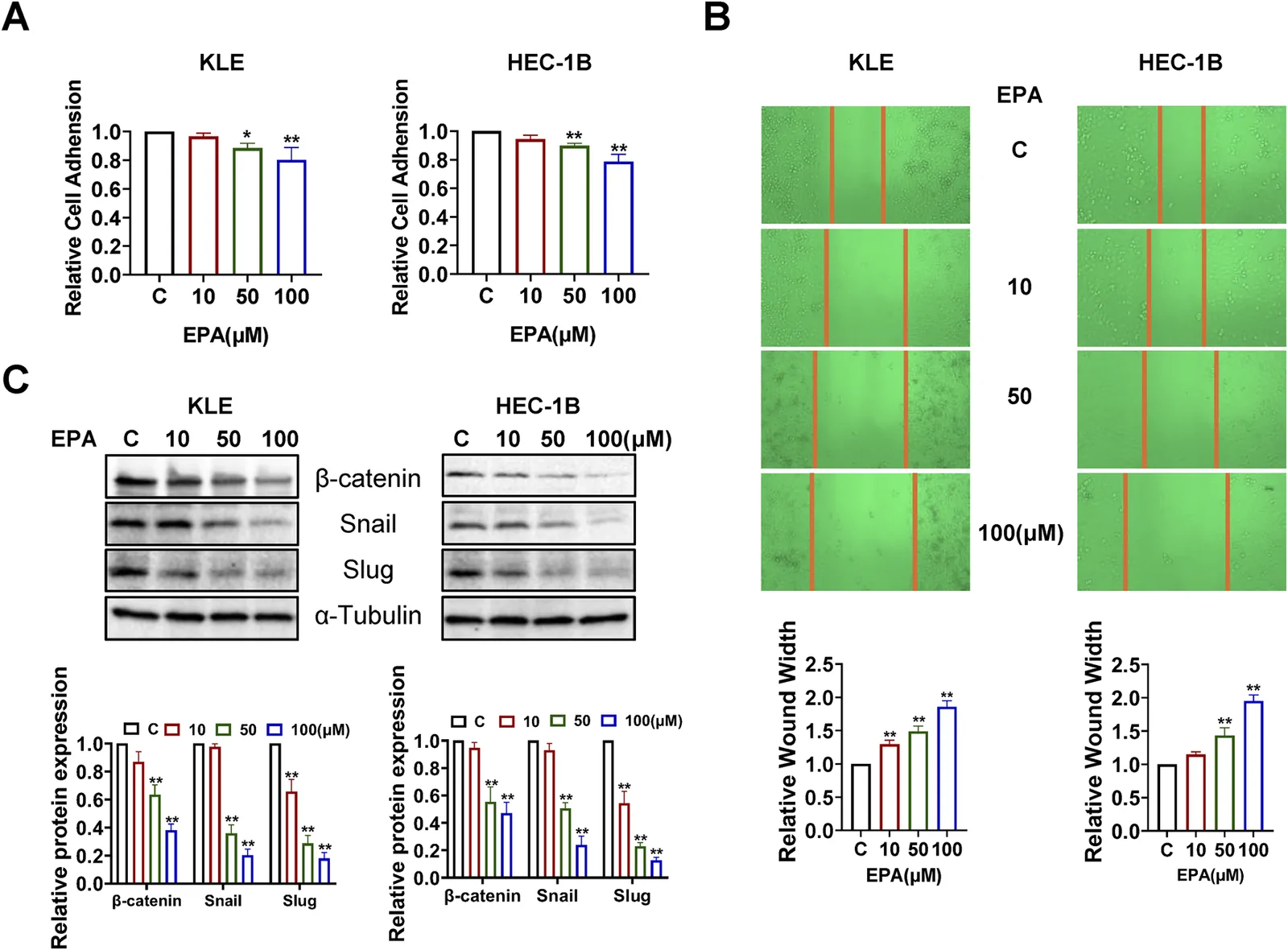
EPA inhibited cell adhesion and invasion in EC cells. EPA effectively reduced the adhesion capabilities of KLE and HEC-1B cells in a dose-dependent manner. **(A)** Wound healing assay revealed that EPA decreased cell migration in both cell lines following 24 h of treatment. **(B)** Western blot analysis showed that EPA treatment for 24 h decreased the expression of β-catenin, Slug, and Snail in both cell lines. **(C)** *p < 0.05, **p < 0.01 compared with control.

### 3.6 EPA reduced the inflammatory response in EC

To determine whether EPA suppresses inflammatory signaling in EC, we first assessed COX-2 expression following 6 h of EPA treatment. Western blot analysis showed that EPA markedly reduced COX-2 levels in both HEC-1B and KLE cells (Figure 6A). Because the NF-κB pathway plays a central role in regulating inflammatory responses, we next examined whether EPA modulates NF-κB activation. As expected, TNF-α stimulation for 30 min robustly induced NF-κB phosphorylation; however, this induction was partially attenuated when cells were pre-treated with EPA for 2 h (Figure 6B). Consistent with these *in vitro* findings, IHC analysis demonstrated that 4 weeks of EPA treatment reduced COX-2 expression in endometrial tumor tissues from both obese and lean *LKB1* ^*fl/fl*^ *p53* ^*fl/fl*^ mice (Figure 6C). Together, these results indicate that EPA effectively suppresses inflammatory responses in EC cells and tumors, at least in part through inhibition of COX-2-mediated signaling and blockade of NF-κB activation.

**FIGURE 6:**
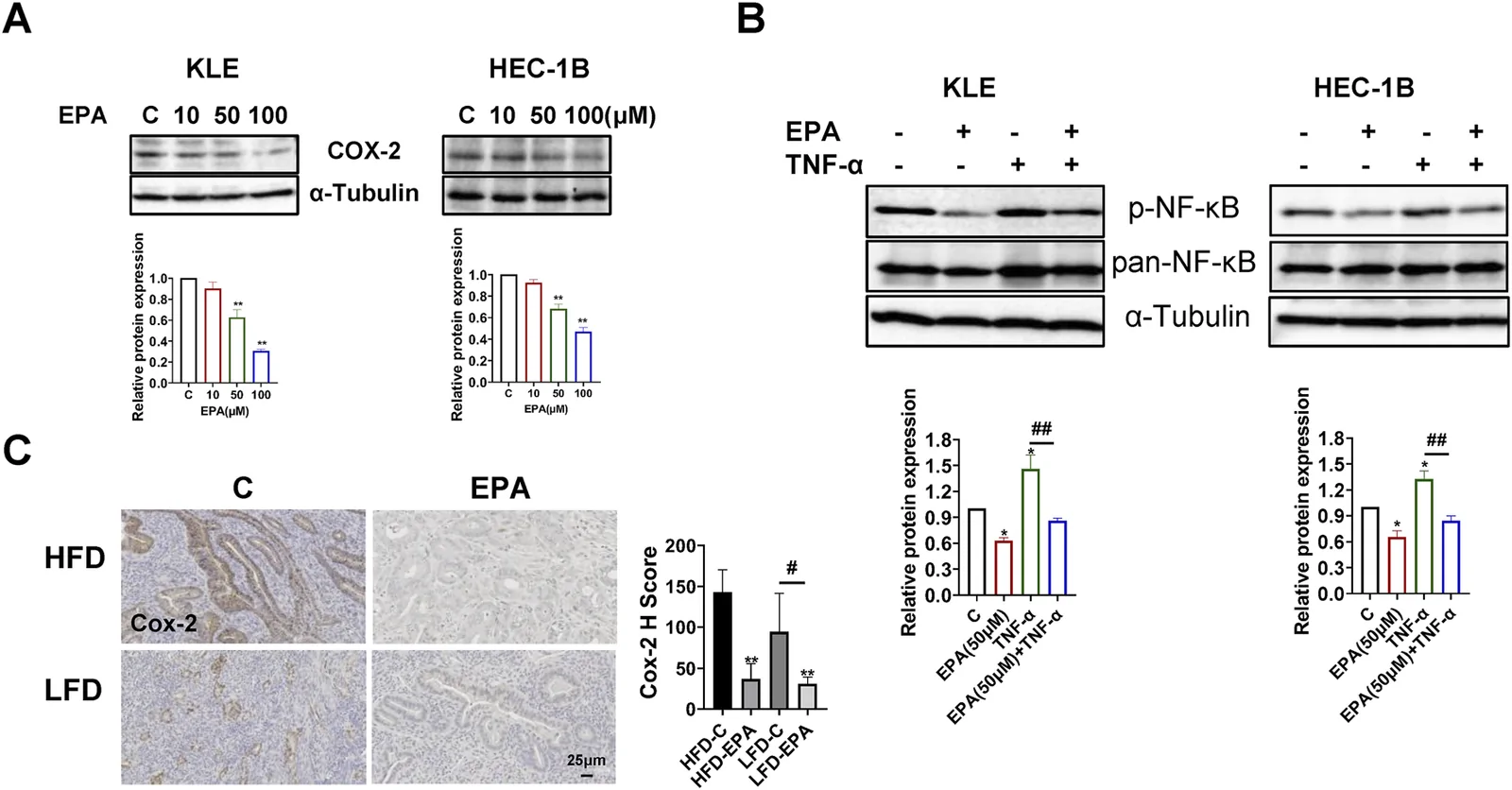
EPA inhibits the expression of COX-2 in EC cells. EPA treatment for 6 h effectively reduced expression of COX-2 in KLE and HEC-1B cells, as shown by Western blot analysis. **(A)** KLE and HEC-1B cells were incubated overnight in medium containing 0.5% charcoal-stripped FBS. Treatment of both cell lines with TNF-α for 30 min significantly increased phosphorylation of NF-κB; however, pretreatment with EPA for 2 h effectively reduced TNF-α-induced levels of phosphorylated NF-κB. **(B)** IHC results revealed that EPA inhibited the expression of COX-2 in EC tissues of *LKB1* ^*fl/fl*^ *p53* ^*fl/fl*^ mice (n = 6). **(C)** *p < 0.05, **p < 0.01 compared with control. #p < 0.05, ##p < 0.01, between groups.

### 3.7 EPA synergistically enhanced carboplatin sensitivity in EC

To determine whether EPA enhances the efficacy of chemotherapeutic agents in EC cells, HEC-1B and KLE cells were treated with combinations of EPA (10, 50, and 100 μM) and carboplatin (10, 25, and 50 μM) for 72 h. MTT assays showed that, at lower concentrations, the combination treatment significantly increased growth inhibition compared with either agent alone in both cell lines (Figure 7A). Consistent with these findings, co-treatment with 50 μM EPA and 25 μM carboplatin for 3 h markedly elevated intracellular ROS levels. In HEC-1B cells, ROS increased by 17.22% with the combination treatment, compared with increases of 8.53% and 7.84% following EPA or carboplatin alone, respectively. In KLE cells, ROS levels increased by 24.69% with the combination, compared with 13.00% and 7.46% for EPA and carboplatin alone (Figure 7B). Caspase-3 activity assays further demonstrated that the combination treatment significantly enhanced cleaved caspase-3 activation relative to either single agent (Figure 7C). In addition, co-treatment with 50 μM EPA and 25 μM carboplatin substantially inhibited cell migration. In HEC-1B cells, wound width was significantly increased by 2.06-fold with the combination treatment, compared with1.59-fold and 1.45-fold by EPA and carboplatin alone, respectively. Similarly, in KLE cells, wound width was significantly increased by 2.09-fold with the combination, compared with 1.55 and 1.67-fold inhibition by EPA and carboplatin alone (Figure 7D). Western blot analysis further revealed that the combination of EPA and carboplatin more effectively increase the expression of PDI, and reduced the expression of Bcl-2, CDK4, and phosphorylated AKT than either agent alone in both cell lines (Figure 7E). Together, these findings indicate that EPA enhances carboplatin sensitivity in EC cells, likely through the induction of oxidative stress and activation of apoptotic signaling pathways.

**FIGURE 7:**
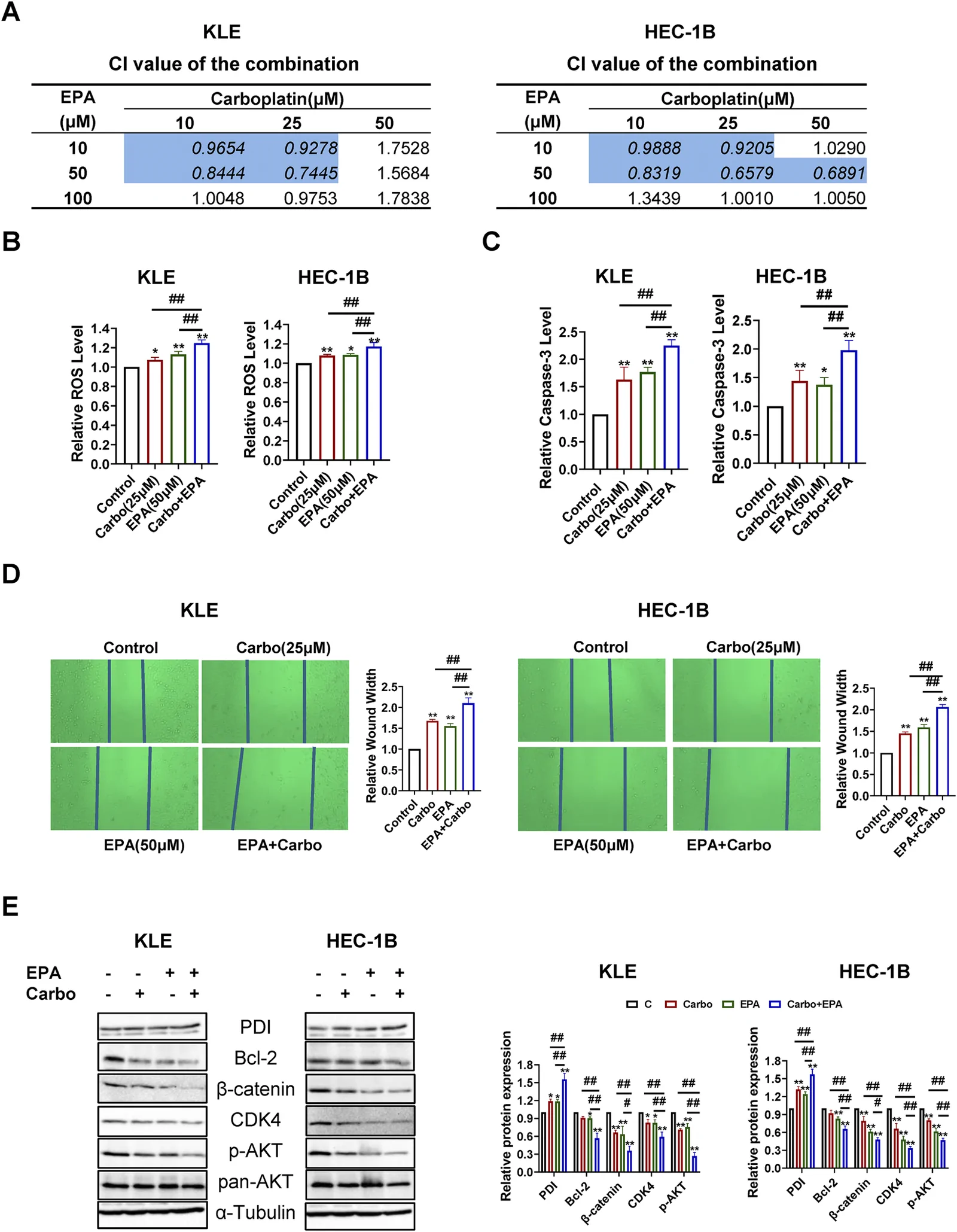
EPA synergistically enhances the sensitivity of EC cells to carboplatin. KLE and HEC-1B cells were co-incubated with EPA, carboplatin, or their combination for 72 h. Based on the MTT assay results, the combination index (CI) for the combination treatment was calculated to assess drug interaction effects. **(A)** The combined treatment induced higher levels of intracellular ROS **(B)** and cleaved caspase-3 activity **(C)** in both cell lines. The combination treatment of EPA and carboplatin exerted a more significant inhibitory effect on cell migration compared with each agent alone in KLE and HEC-1B cells. **(D)** Western blot analysis was used to analyze the effect of EPA, carboplatin, or their combination on the expression of PDI, BcL-2, β-catenin, CDK4, and phosphorylated-AKT after 3–24 h of treatment in both cell lines. **(E)** *p < 0.05, **p < 0.01 compared with control. #p < 0.05, ##p < 0.01, between groups.

## 4 Discussion

Although polyunsaturated fatty acids (PUFAs) are not currently approved by the FDA for cancer therapy, accumulating pre-clinical evidence indicates that they exert anti-cancer effects by modulating multiple pathways involved in tumor initiation and progression, including cell proliferation, survival, angiogenesis, inflammation, metastasis, and epigenetic regulation (Rahman et al., 2013; Freitas and Campos, 2019; Akbar et al., 2024). EC is characterized by a strong interplay between metabolic dysregulation and chronic inflammation, both of which contribute to its pathogenesis and progression (Hooks et al., 2025; Buza et al., 2026). In this study, we demonstrate that EPA reduces cell viability and colony formation in a dose-dependent manner across multiple EC cell lines. *In vivo*, 4 weeks of EPA treatment significantly inhibited endometrial tumor growth in both obese and lean *LKB1* ^*fl/fl*^ *p53* ^*fl/fl*^ mice, accompanied by decreased expression of Ki-67, COX-2, and phosphorylated S6 in tumor tissues. Mechanistically, the anti-tumor effects of EPA involve multiple processes, including induction of G1-phase cell-cycle arrest, promotion of cellular stress and apoptosis, inhibition of cell adhesion and invasion, and suppression of inflammatory signaling. Moreover, EPA enhances the sensitivity of EC cells to carboplatin, resulting in greater inhibition of proliferation, reduced invasion, and increased apoptosis compared with either agent alone. Collectively, these findings support EPA as a biologically active compound with potential therapeutic value in the treatment of EC.

EPA exerts its anti-tumor effects, at least in part, through modulation of key oncogenic signaling pathways, including the PI3K/AKT/mTOR and MAPK cascades. Prior studies have shown that EPA and other long-chain n-3 PUFAs suppress AKT/mTOR signaling, resulting in reduced phosphorylation of downstream effectors, such as S6, and subsequent inhibition of protein synthesis and cell proliferation across multiple cancer types (Friedrichs et al., 2011; Andrade-Vieira et al., 2013; West et al., 2020). Consistent with these findings, our data demonstrate that EPA markedly decreased the expression of phosphorylated AKT and phosphorylated S6 in EC cell lines and reduced phosphorylated S6 expression in endometrial tumors. Interestingly, EPA increased phosphorylated ERK1/2 in EC cells. This likely reflects a compensatory feedback mechanism in which inhibition of the PI3K/AKT/mTOR axis relieves negative regulatory loops, leading to activating the MAPK/ERK pathway, a phenomenon reported in several cancer models following mTOR inhibition (Serini and Calviello, 2017; Roze et al., 2014). Importantly, in the context of EPA treatment, ERK activation may not promote proliferation but instead contribute to cellular stress responses or apoptosis (Roze et al., 2014; Roskoski, 2012). However, the precise role of ERK activation in mediating the effects of EPA, and whether it represents an adaptive cellular response to EPA treatment, remains to be elucidated. Consistent with these pathway alterations, EPA significantly increased intracellular ROS levels and reduced mitochondrial membrane potential, indicating induction of mitochondrial dysfunction and oxidative stress. Excessive oxidative stress disrupts mitochondrial integrity and activates stress-responsive signaling pathways. Mitochondrial dysfunction promotes the release of pro-apoptotic factors such as cytochrome c and facilitates caspase activation, linking oxidative stress to apoptosis (Ralph et al., 2010; Vostrikova et al., 2020). In line with this mechanism, EPA robustly activated cleaved caspase-3, -8 and -9, while reducing the expression of the anti-apoptotic proteins MCl-1, Bcl-xL, and Bcl-2, confirming involvement of both the intrinsic and extrinsic apoptotic pathways. Importantly, 4 weeks of EPA treatment decreased Ki-67 and Bcl-xL expression in endometrial tumors from both obese and lean in *LKB1* ^*fl/fl*^ *p53* ^*fl/fl*^ mice, further supporting the role of EPA-induced apoptosis in suppressing tumor proliferation. Collectively, these coordinated effects, including inhibition of pro-survival AKT/mTOR signaling, differential modulation of MAPK pathways, induction of oxidative and mitochondrial stress, and activation of apoptotic cascades, shift the balance from cell survival toward growth inhibition and apoptosis, ultimately contributing to the anti-tumor activity of EPA in EC.

In our study, EPA treatment significantly reduced cell adhesion and migration, accompanied by decreased expression of the EMT-associated transcription factors β-catenin, Slug, and Snail. These findings are consistent with our previous observations that EPA suppresses the invasive capacity of uterine serous carcinoma cells, further supporting a role for EPA in limiting metastatic progression in EC (Zhang et al., 2026). Because EMT is tightly regulated by oncogenic signaling pathways, such as PI3K/AKT/mTOR and MAPK, the suppression of β-catenin, Slug, and Snail observed here may be mechanistically linked to EPA-mediated inhibition of AKT signaling and modulation of MAPK activity (Xu et al., 2015; Inan and Hayran, 2019). Inhibition of the AKT pathway is known to downregulate EMT programs, while stress-associated ERK activation may further impair cellular plasticity and invasive behavior (Li et al., 2016; Shin et al., 2019). Moreover, EPA-induced oxidative stress and apoptosis may additionally disrupt cytoskeletal organization and cell–matrix interactions, thereby limiting adhesion and invasion (Jalmukhambetova et al., 2025; Silva and Colquhoun, 2025). Overall, our findings indicate that EPA suppresses cell adhesion and invasion in EC, at least in part through modulation of EMT-associated signaling pathways.

Chronic inflammation is a well-established driver of EC development and progression, particularly in the context of obesity-associated metabolic dysregulation and a persistently activated tumor-promoting microenvironment (Tan et al., 2025). Pro-inflammatory cytokines, such as TNF-α, activate oncogenic signaling pathways, including NF-κB, leading to induction of COX-2 and subsequent production of prostaglandin E_2_ (PGE_2_), which collectively promote tumor cell proliferation, survival, invasion, and the EMT process (Wang and Dubois, 2010). EPA is well recognized for its potent anti-inflammatory properties, in part through inhibition of NF-κB activation and suppression of pro-inflammatory lipid mediators (Irún et al., 2019; Calder, 2010). In addition to its direct biological effects, EPA can be enzymatically converted into bioactive lipid mediators, including 18-hydroxyeicosapentaenoic acid (18-HEPE) and E-series resolvins such as resolvin E1 (RvE1), RvE2, and RvE3, which possess anti-inflammatory and pro-resolving activities and may contribute to the regulation of tumor-associated inflammation (Arita et al., 2005; Kiyasu et al., 2024; Weylandt et al., 2012). Thus, the biological effects of EPA may reflect both direct actions of EPA and the activities of its downstream lipid mediators. Importantly, the effects of EPA in obesity-associated cancer may be context dependent and may not necessarily parallel changes in systemic metabolic or inflammatory status (Inoue-Yamauchi et al., 2018). EPA may therefore exert anti-tumor effects through direct actions on tumor cells and/or local modulation of the tumor microenvironment, even in the absence of substantial changes in obesity-associated systemic inflammation (Wang et al., 2014; Ishii et al., 2009). Consistent with this possibility, in the present study, EPA reduced basal COX-2 expression in endometrial tumors from both obese and lean mice and markedly attenuated TNF-α–induced upregulation of COX-2 and phosphorylated NF-κB in EC cells. These findings suggest that EPA may suppress a tumor-promoting inflammatory program involving the TNF-α/NF-κB/COX-2 axis, thereby limiting inflammatory signaling that supports tumor progression and may contribute to chemoresistance. However, because EPA-derived lipid mediators were not directly measured in the present study, we cannot determine whether these effects are mediated exclusively by EPA itself or partly through its downstream metabolites. Further studies evaluating EPA metabolism and the contribution of specific EPA-derived mediators will be important for defining the mechanisms underlying the anti-tumor effects of EPA in EC.

Accumulating evidence indicates that EPA enhances the efficacy of chemotherapeutic agents, including 5-fluorouracil, cisplatin, temozolomide (TMZ), and doxorubicin, by inhibiting cell proliferation, inducing apoptosis, and suppressing invasion across multiple cancer types (Silva and Colquhoun, 2025; Gurav et al., 2023; Ogo et al., 2021; Dupertuis et al., 2021). Consistent with these observations, our previous work demonstrated that EPA synergistically increased carboplatin sensitivity in uterine serous carcinoma cells, resulting in enhanced inhibition of cell proliferation and migration. In the present study, co-treatment with EPA and carboplatin produced significantly greater inhibition of cell proliferation than either agent alone, with combination index (CI) analysis confirming a synergistic interaction. This enhanced anti-proliferative effect was accompanied by increased intracellular ROS generation and augmented activation of cleaved caspase-3. The combination treatment also more effectively inhibited cell migration in both KLE and HEC-1B cells, suggesting that EPA may potentiate carboplatin’s ability to suppress invasive behavior in EC. Mechanistically, Western blot analysis revealed that the combination treatment resulted in more pronounced downregulation of phosphorylated AKT, Bcl-2, β-catenin, and CDK4, highlighting synergistic inhibition of pro-survival signaling, cell cycle progression, and the Wnt/β-catenin-mediated pathway. These molecular alterations may increase the susceptibility of EC cells to oxidative stress, disrupt mitochondrial integrity, and amplify caspase activation, thereby further potentiating cell death (Ralph et al., 2010; Vostrikova et al., 2020). Together, these findings suggest that EPA enhances carboplatin sensitivity through integrated modulation of AKT signaling, ROS-mediated stress, apoptotic pathways, and cell-cycle regulation, supporting its potential as an adjuvant therapeutic strategy to improve outcomes in endometrial cancer. An important consideration for the translational relevance of our findings is whether the EPA concentrations used *in vitro* are achievable in humans. Human pharmacokinetic studies indicate that oral EPA administration can substantially increase circulating EPA concentrations in a dose- and formulation-dependent manner. For example, following administration of icosapent ethyl at 4 g/day for 28 days, mean total plasma EPA concentrations increased from approximately 19 μg/mL at baseline to a maximum concentration of 366 μg/mL (approximately 1.2 mM) at 5 h after dosing, and clinical studies have reported end-of-treatment plasma EPA concentrations exceeding 170 μg/mL following 4 g/day of icosapent ethyl (Braeckman et al., 2014; Bays et al., 2016). The highest EPA concentration used in our *in vitro* experiments was 100 μM (approximately 30 μg/mL), which is below these reported total plasma EPA concentrations. However, this comparison should be interpreted cautiously because total plasma EPA does not directly reflect the concentration of free EPA available to tumor cells, and comparable concentrations within tumor tissue cannot be inferred from plasma exposure alone. Importantly, our findings are further supported by the *in vivo* efficacy of EPA, as daily oral administration of EPA at 50 mg/kg for 4 weeks significantly inhibited tumor growth in both lean and obese mice without significant changes in body weight. Although these findings provide *in vivo* support for the antitumor activity of EPA observed in our cell-based experiments, the 50 mg/kg dose represents a pharmacological exposure and should not be directly equated with nutritional EPA intake in humans. Dietary EPA intake and conventional supplementation generally result in lower and more variable systemic exposure than high-dose pharmaceutical EPA preparations. Collectively, these findings support the potential translational relevance of EPA as an antitumor agent while underscoring the need for further pharmacokinetic and pharmacodynamic studies to define the relationship between systemic EPA exposure, tumor EPA accumulation, and antitumor efficacy.

Several limitations of the present study should also be acknowledged. Although EPA sensitivity was initially evaluated across five EC cell lines and its antitumor activity was further demonstrated in a hormone-sensitive, ER-positive *in vivo* model, the mechanistic studies were primarily performed in HEC-1B and KLE cells, which represent relatively aggressive EC models with distinct molecular backgrounds. Thus, mechanistic validation was not systematically performed across hormone-sensitive models or molecularly defined subgroups, including PTEN-deficient and TP53-abnormal EC, and whether EPA responses and mechanisms vary according to molecular subtype or genetic background remains to be determined. In addition, the present study did not directly assess whether EPA alters carboplatin uptakes, intracellular platinum accumulation or distribution, platinum-DNA adduct formation, or the expression or activity of platinum transport and resistance pathways. Thus, the extent to which changes in platinum disposition contribute to the enhanced carboplatin sensitivity observed with EPA combination treatment remains unclear. Future studies using additional molecularly characterized EC cell lines and animal models will be important to determine whether the anti-tumor effects and mechanisms of EPA vary according to molecular and genomic background. Further investigation of intracellular platinum accumulation, platinum-DNA adduct formation, and relevant platinum transport and resistance mechanisms, together with evaluation of potential changes in cellular membrane composition, may help clarify the mechanisms underlying EPA-mediated carboplatin sensitization.

In conclusion, omega-3 PUFAs, particularly DHA and EPA, have been evaluated in multiple clinical studies over the past decade for their potential roles in cancer prevention and treatment. Several studies suggest that EPA, when used as an adjunct therapy, is safe and may beneficially modulate tumor biology, inflammation, and treatment tolerance (Molfino et al., 2026; Freitas and Campos, 2019; Sun et al., 2025; Patursson et al., 2021; Ueno et al., 2022). Our findings clearly demonstrate that EPA exerts potent anti-tumor effects through coordinated regulation of multiple oncogenic processes in EC cells. EPA synergistically enhanced the efficacy of carboplatin, leading to greater inhibition of cell proliferation and invasion through modulation of ROS generation, activation of apoptotic pathways, suppression of EMT process, and inhibition of the AKT/mTOR signaling pathway. EPA also reduced COX-2 expression and attenuated TNF-α-induced phosphorylation of NF-κB, underscoring its anti-inflammatory activity. Importantly, 4 weeks of EPA treatment significantly reduced endometrial tumor growth in both obese and lean conditions *in vivo*. Together, these results support the potential of EPA as an adjunct therapeutic agent for EC and provide a strong rationale for future clinical evaluation in patients with EC. However, further preclinical and translational studies are warranted to validate these findings and determine whether the therapeutic potential of EPA can be translated into clinical benefit in patients with EC.

## Data Availability

The original contributions presented in the study are included in the article/Supplementary Material, further inquiries can be directed to the corresponding authors.
