# Supplementary material for: Eicosapentaenoic acid exhibits antitumor effects and synergistically enhances carboplatin sensitivity in endometrial cancer
Source: Front Pharmacol. 2026 Oct 21;17:1960634. doi: 10.3389/fphar.2026.1960634 (PMC13732774; doi:10.3389/fphar.2026.1960634)
Supplement: Supplementary file 1 [file DataSheet1.pdf]

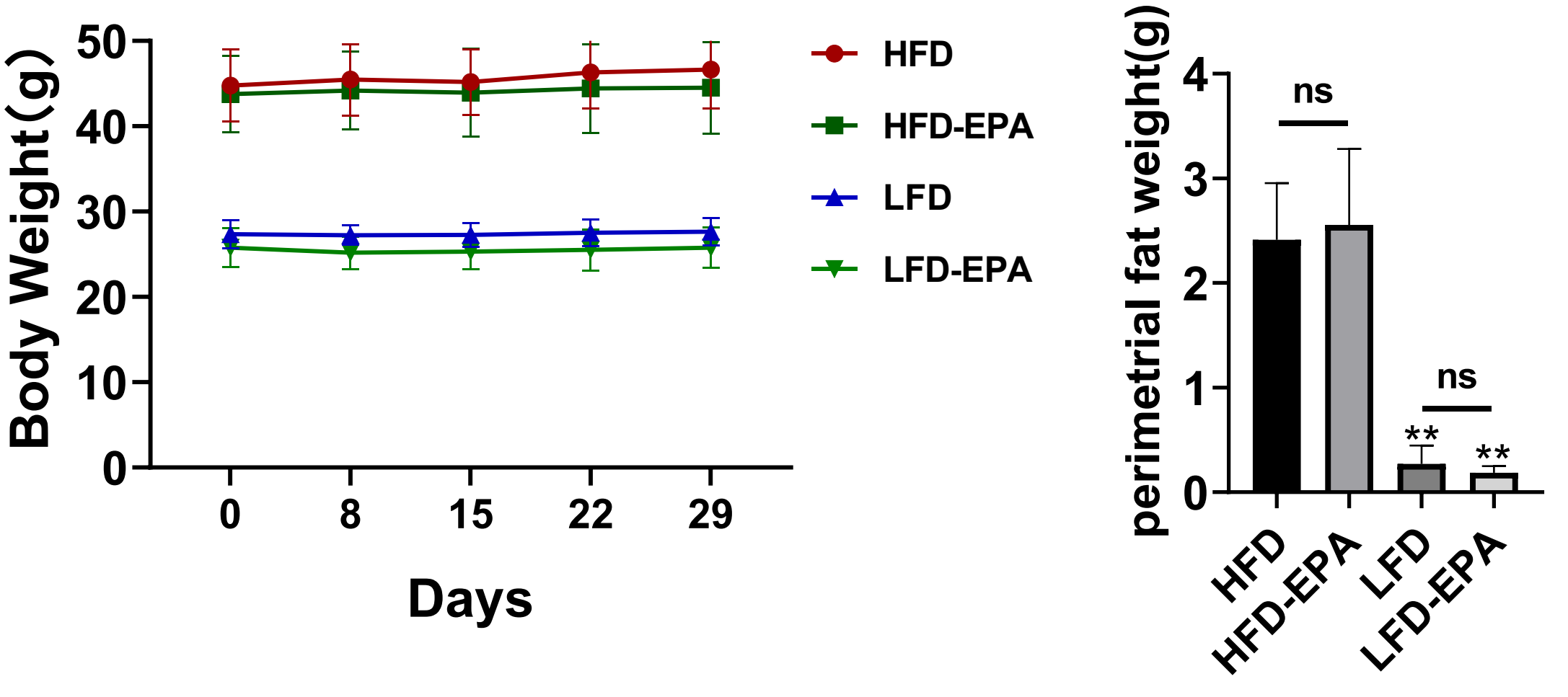
Body Weight(g)
50
40
30
20
10
0
0
8
15
22
29
Days
HFD
HFD-EPA
LFD
LFD-EPA
perimetrial fat weight(g)
4
3
2
1
0
ns
ns
**
**
HFD
HFD-EPA
LFD
LFD-EPA
